# Three-dimensional network in piper­azine-1,4-diium–picrate–piperazine (1/2/1)

**DOI:** 10.1107/S1600536808005710

**Published:** 2008-03-05

**Authors:** Zhong-Long Wang, Li-Hui Jia

**Affiliations:** a Science College, Three-Gorges University, Yichang 443002, People’s Republic of China; b School of Chemical Engineering and Pharmacy, Wuhan Institute of Chemical Technology, Wuhan 430074, People’s Republic of China

## Abstract

In the title compound, C_4_H_12_N_2_^2+^·2C_6_H_2_N_3_O_7_^−^·C_4_H_10_N_2_, the piperazine-1,4-diium cations and piperazine mol­ecules lie on crystallographic inversion centres. In the crystal structure, inter­molecular N—H⋯O and N—H⋯N hydrogen bonds link the components to form two-dimensional layers parallel to the (001) plane. These layers are, in turn, connected by weak inter­molecular C—H⋯O hydrogen bonds and π–π stacking inter­actions [centroid–centroid distance between parallel aryl rings = 3.764 (2) Å, interplanar spacing = 3.500 (2) Å and ring offset = 1.387 (2) Å], forming a three-dimensional framework.

## Related literature

For related literature, see: Akutagawa *et al.* (2003 ▶); Anitha *et al.* (2006*a* ▶,*b* ▶); Arnaud *et al.* (2007 ▶); Colquhoun *et al.* (1986 ▶); Hundal *et al.* (1997 ▶); Kavitha *et al.* (2005 ▶, 2006 ▶); Ma *et al.* (2005 ▶); Szumna *et al.* (2000 ▶); Vembu *et al.* (2003 ▶). 
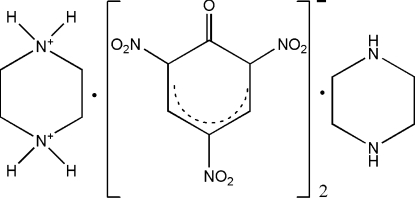


## Experimental

### 

#### Crystal data

C_4_H_12_N_2_^2+^·2C_6_H_2_N_3_O_7_^−^·C_4_H_10_N_2_
*M* *_r_* = 630.50
Triclinic, 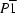

*a* = 7.7150 (6) Å
*b* = 8.1658 (6) Å
*c* = 11.3024 (8) Å
α = 98.140 (1)°
β = 98.974 (1)°
γ = 109.250 (1)°
*V* = 649.62 (8) Å^3^
*Z* = 1
Mo *K*α radiation
μ = 0.14 mm^−1^
*T* = 299 (2) K
0.20 × 0.10 × 0.06 mm

#### Data collection

Bruker SMART APEX CCD area-detector diffractometer
Absorption correction: multi-scan (*SADABS*; Sheldrick, 1997 ▶) *T*_min_ = 0.963, *T*_max_ = 0.992
6131 measured reflections
2258 independent reflections
1917 reflections with *I* > 2σ(*I*)
*R*_int_ = 0.028

#### Refinement

*R*[*F* ^2^ > 2σ(*F* ^2^)] = 0.068
*wR*(*F* ^2^) = 0.169
*S* = 1.13
2258 reflections
208 parameters
H atoms treated by a mixture of independent and constrained refinement
Δρ_max_ = 0.27 e Å^−3^
Δρ_min_ = −0.27 e Å^−3^

### 

Data collection: *SMART* (Bruker, 2001 ▶); cell refinement: *SAINT-Plus* (Bruker, 2001 ▶); data reduction: *SAINT-Plus*; program(s) used to solve structure: *SHELXS97* (Sheldrick, 2008 ▶); program(s) used to refine structure: *SHELXL97* (Sheldrick, 2008 ▶); molecular graphics: *PLATON* (Spek, 2003 ▶); software used to prepare material for publication: *PLATON*.

## Supplementary Material

Crystal structure: contains datablocks global, I. DOI: 10.1107/S1600536808005710/lh2598sup1.cif

Structure factors: contains datablocks I. DOI: 10.1107/S1600536808005710/lh2598Isup2.hkl

Additional supplementary materials:  crystallographic information; 3D view; checkCIF report

## Figures and Tables

**Table 1:** Hydrogen-bond geometry (Å, °)

*D*—H⋯*A*	*D*—H	H⋯*A*	*D*⋯*A*	*D*—H⋯*A*
N4—H4*A*⋯O1	0.86 (4)	1.95 (4)	2.745 (4)	153 (4)
N4—H4*A*⋯O7	0.86 (4)	2.31 (4)	2.870 (5)	123 (3)
N4—H4*B*⋯N5	0.86 (4)	1.94 (4)	2.799 (4)	176 (4)
N5—H5*A*⋯O2^i^	0.86 (4)	2.41 (4)	3.153 (5)	145 (4)
C2—H2⋯O6^ii^	0.93	2.47	3.335 (5)	155
C7—H7*B*⋯O1^iii^	0.97	2.52	3.211 (5)	128
C8—H8*B*⋯O1	0.97	2.60	3.267 (5)	127
C8—H8*B*⋯O2	0.97	2.52	3.458 (5)	162
C9—H9*A*⋯O5^iv^	0.97	2.60	3.272 (5)	127
C10—H10*A*⋯O4^v^	0.97	2.52	3.310 (5)	138

Symmetry codes: (i) 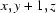; (ii) 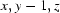; (iii) 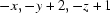; (iv) 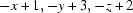; (v) 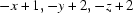.

**Fig. 1.**
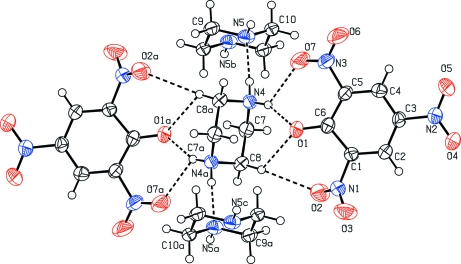
Molecular structure of (I), showing the atom-numbering scheme. Displacement ellipsoids are drawn at the 50% probability level. Inter-ion hydrogen bonds are shown as dashed lines, atoms marked with 'a', 'b' and 'c' are at symmetry positions (-x, 2 - y, 1 - z), (1 - x, 3 - y, 1 - z) and (-1 + x, -1 + y, z), respectively. An additional piperazine molecule is shown to illustrate the hydrogen bonding.

**Fig. 2.**
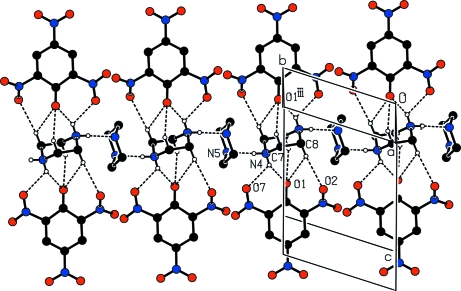
Part of the crystal structure of (I), showing the formation of the one-dimensional tape running parallel to the [110] direction. Hydrogen bonds are shown as dashed lines. For the sake of clarity, H atoms not involved in the motifs have been omitted. [symmetry code: (iii) -x, -y + 2, -z + 1]

**Fig. 3.**
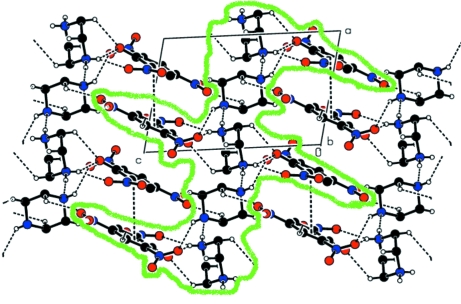
Part of the crystal structure of (I), showing the linkage of adjacent 2-D layers by C9—H9A···O5, C10—H10A···O4 hydrogen bonds and π-π stacking interactions, which form the three-dimensional network. The green outlined area shows the 2-D layer parallel to the (001) plane. For the sake of clarity, H atoms not involved in the motifs have been omitted.

## supplementary crystallographic information

### Comment

Studies of picric acid (abbr. PA, p*K*a = 0.38) have been carried out for many years due to its formation of salts which involve electrostatic forces, multiple hydrogen bond modes (*e.g.* Hundal *et al.*, 1997; Szumna *et al.*, 2000) and π–π stacking interactions (Colquhoun *et al.*, 1986) which can improve the quality of the crystalline materials. Recently, picrate anion containing molecular adducts have also been reported frequently in order to probe the competition between various intermolecular forces in crystal engineering (Anitha *et al.*, 2006*a*, 2006*b*; Vembu *et al.*, 2003; Ma *et al.*, 2005; Akutagawa *et al.*, 2003, Kavitha *et al.*, 2005; Arnaud *et al.*, 2007). As part of our study on molecular adducts involved with PA and piperazine (abbr. PP), we report here the molecular and supra-molecular structure of the title compound (I).

In (I), the asymmetric unit (atoms labelled without lower case suffixes in Fig.1) consists of one picrate anion, half a PA di-cation and half a neutral PA molecule. In the picrate anion, the nitro group at the 4-position is almost coplanar with the phenyl ring with a dihedral angle of only 4.1 (2)°, however, the nitro groups at the 2- and 6-positions are both significantly twisted out of the plane of the benzene ring, with dihedral angles of 45.2 (2)° and 21.7 (2)°, respectively. The rotations of the nitro groups at the 2- and 6- positions means that the picrate anion retains the approximate mirror symmetry which is also observed in the structure of a recently reported analog (Kavitha *et al.*, 2006).

Analysis of the crystal packing of (I) shows that the component ions and molecules are linked into a simple three-dimensional network by a combination of N–H···O (or N), C–H···O hydrogen bonds and π–π stacking interactions which can be analyzed in terms of several substructures. First, by the co-operative hydrogen-bonding actions, *i.e.* bifurcated N4···O1(O7), bifurcated C8···O1(O2) and C7···O1 (-*x*, 2 - *y*, 1 - *z*) hydrogen bonds, the PA anions, PP di-cations and PP neutral molecules are linked into a one-dimensional tape structure parallel to the [110] direction generated by translation and inversion operations (Fig.2). Secondly, by a combinative actions of N5—H5A···O2 (*x*, 1 + *y*, *z*) and C2—H2···O6 (*x*, *y* - 1, *z*) hydrogen-bonds, the adjacent [110] 1-D tapes are joined together, forming a two-dimensional layer parallel to the (001) plane lying in domain of -0.299 < *z* < 1.299 (Fig.3). Finally, the neighbouring (001) layers are joined together by means of C9···O5 (-*x* + 1, -*y* + 3, -*z* + 2), C10···O4 (-*x* + 1, -*y* + 2, -*z* + 2) hydrogen bonds and π–π stacking interactions, which form the simple 3-D network. The geometry details of the π–π stacking interactions are as follows. The C1—C6 aryl rings of the anions at (*x*, *y*, *z*) and (1 - *x*, 2 - *y*, 2 - *z*) are strictly parallel, with an inter-planar spacing of 3.500 (2) Å; the ring-centroid separation is 3.764 (2) Å, corresponding to a ring offset of 1.387 (2) Å.

### Experimental

All the reagents and solvents were used as obtained without further purification. 1:2 molar amount of anhydrous piperazine (0.2 mmol, 17.2 mg) and picric acid (0.4 mmol, 91.6 mg) were dissolved in 95% methanol (10 ml). The mixture was stirred for half an hour at ambient temperature and then filtered. The resulting yellow solution was kept in air for several days. Plate yellow crystals of (I) suitable for single-crystal X-ray diffraction analysis were grown by slow evaporation of the solution at the bottom of the vessel (yield: 45%, 56.7 mg, based on 2:1 organic salt; melting point: 512–514 K).

### Refinement

H atoms bonded to C atoms were placed in calculated positions with C–H=0.93Å (aromatic), 0.97Å (methylene) and *U*_iso_(H) = 1.2*U*_eq_(both aromatic and methylene C). H atoms attached to N atoms were located from the difference maps with the N–H distances being refined freely and *U*_iso_(H) =1.2*U*_eq_(N).

### Crystal data

**Table:**

C_4_H_12_N_2_^2+^·2C_6_H_2_N_3_O_7_^–^·C_4_H_10_N_2_	*Z* = 1
*M**_r_* = 630.50	*F*_000_ = 328
Triclinic, *P*1	*D*_x_ = 1.612 Mg m^−^^3^
Hall symbol: -P 1	Mo *K*α radiation λ = 0.71073 Å
*a* = 7.7150 (6) Å	Cell parameters from 2518 reflections
*b* = 8.1658 (6) Å	θ = 2.7–26.2º
*c* = 11.3024 (8) Å	µ = 0.14 mm^−^^1^
α = 98.140 (1)º	*T* = 299 (2) K
β = 98.974 (1)º	Plate, yellow
γ = 109.250 (1)º	0.20 × 0.10 × 0.06 mm
*V* = 649.62 (8) Å^3^	

### Data collection

**Table:**

Bruker SMART APEX CCD area-detector diffractometer	2258 independent reflections
Radiation source: fine focus sealed Siemens Mo tube	1917 reflections with *I* > 2σ(*I*)
Monochromator: graphite	*R*_int_ = 0.028
*T* = 299(2) K	θ_max_ = 25.0º
0.3° wide ω exposures scans	θ_min_ = 2.7º
Absorption correction: multi-scan(SADABS; Sheldrick, 1997)	*h* = −9→9
*T*_min_ = 0.963, *T*_max_ = 0.992	*k* = −9→9
6131 measured reflections	*l* = −13→13

### Refinement

**Table:**

Refinement on *F*^2^	Secondary atom site location: difference Fourier map
Least-squares matrix: full	Hydrogen site location: inferred from neighbouring sites
*R*[*F*^2^ > 2σ(*F*^2^)] = 0.068	H atoms treated by a mixture of independent and constrained refinement
*wR*(*F*^2^) = 0.169	*w* = 1/[σ^2^(*F*_o_^2^) + (0.0397*P*)^2^ + 1.340*P*] where *P* = (*F*_o_^2^ + 2*F*_c_^2^)/3
*S* = 1.13	(Δ/σ)_max_ < 0.001
2258 reflections	Δρ_max_ = 0.27 e Å^−^^3^
208 parameters	Δρ_min_ = −0.27 e Å^−^^3^
Primary atom site location: structure-invariant direct methods	Extinction correction: none

### Special details

**Table:**

Geometry. All e.s.d.'s (except the e.s.d. in the dihedral angle between two l.s. planes) are estimated using the full covariance matrix. The cell e.s.d.'s are taken into account individually in the estimation of e.s.d.'s in distances, angles and torsion angles; correlations between e.s.d.'s in cell parameters are only used when they are defined by crystal symmetry. An approximate (isotropic) treatment of cell e.s.d.'s is used for estimating e.s.d.'s involving l.s. planes.
Refinement. Refinement of *F*^2^ against ALL reflections. The weighted *R*-factor *wR* and goodness of fit *S* are based on *F*^2^, conventional *R*-factors *R* are based on *F*, with *F* set to zero for negative *F*^2^. The threshold expression of *F*^2^ > σ(*F*^2^) is used only for calculating *R*-factors(gt) *etc*. and is not relevant to the choice of reflections for refinement. *R*-factors based on *F*^2^ are statistically about twice as large as those based on *F*, and *R*- factors based on ALL data will be even larger.

### Fractional atomic coordinates and isotropic or equivalent isotropic displacement parameters (Å^2^)

**Table:**

	*x*	*y*	*z*	*U*_iso_*/*U*_eq_	
C1	0.1576 (5)	0.8052 (4)	0.9002 (3)	0.0332 (8)	
C2	0.2353 (5)	0.7965 (5)	1.0152 (3)	0.0378 (9)	
H2	0.2296	0.6883	1.0348	0.045*	
C3	0.3230 (5)	0.9530 (5)	1.1019 (3)	0.0348 (8)	
C4	0.3317 (5)	1.1141 (5)	1.0738 (3)	0.0380 (9)	
H4	0.3912	1.2181	1.1329	0.046*	
C5	0.2518 (5)	1.1200 (5)	0.9578 (3)	0.0371 (8)	
C6	0.1526 (5)	0.9660 (5)	0.8609 (3)	0.0333 (8)	
C7	0.1883 (5)	1.0168 (5)	0.4896 (3)	0.0392 (9)	
H7A	0.3073	1.0068	0.5226	0.047*	
H7B	0.1971	1.0570	0.4132	0.047*	
C8	0.0332 (5)	0.8378 (5)	0.4654 (3)	0.0381 (9)	
H8A	0.0549	0.7561	0.4034	0.046*	
H8B	0.0337	0.7912	0.5397	0.046*	
C9	0.3474 (5)	1.5596 (5)	0.4793 (4)	0.0405 (9)	
H9A	0.4124	1.6869	0.4938	0.049*	
H9B	0.2133	1.5352	0.4581	0.049*	
C10	0.5940 (5)	1.5299 (5)	0.6247 (3)	0.0386 (9)	
H10A	0.6210	1.4848	0.6976	0.046*	
H10B	0.6639	1.6566	0.6425	0.046*	
N1	0.0732 (4)	0.6390 (4)	0.8092 (3)	0.0405 (8)	
N2	0.4143 (5)	0.9504 (4)	1.2226 (3)	0.0443 (8)	
N3	0.2705 (6)	1.2965 (4)	0.9349 (3)	0.0528 (9)	
N4	0.1521 (4)	1.1476 (4)	0.5768 (3)	0.0364 (7)	
H4A	0.157 (6)	1.118 (5)	0.647 (4)	0.044*	
H4B	0.228 (6)	1.255 (6)	0.584 (4)	0.044*	
N5	0.3922 (5)	1.4953 (4)	0.5905 (3)	0.0383 (7)	
H5A	0.359 (6)	1.548 (5)	0.649 (4)	0.046*	
O1	0.0655 (4)	0.9638 (4)	0.7581 (2)	0.0471 (7)	
O2	0.1103 (5)	0.6367 (4)	0.7077 (3)	0.0579 (8)	
O3	−0.0265 (5)	0.5088 (4)	0.8379 (3)	0.0720 (10)	
O4	0.4179 (6)	0.8106 (4)	1.2447 (3)	0.0925 (14)	
O5	0.4870 (5)	1.0892 (4)	1.2985 (3)	0.0599 (9)	
O6	0.3035 (6)	1.4152 (4)	1.0227 (3)	0.0831 (12)	
O7	0.2583 (6)	1.3203 (4)	0.8304 (3)	0.0799 (12)	

### Atomic displacement parameters (Å^2^)

**Table:**

	*U*^11^	*U*^22^	*U*^33^	*U*^12^	*U*^13^	*U*^23^
C1	0.0349 (19)	0.0294 (18)	0.0331 (19)	0.0089 (15)	0.0063 (15)	0.0074 (15)
C2	0.044 (2)	0.0331 (19)	0.036 (2)	0.0137 (17)	0.0052 (17)	0.0123 (16)
C3	0.041 (2)	0.039 (2)	0.0265 (18)	0.0153 (16)	0.0073 (15)	0.0104 (15)
C4	0.043 (2)	0.0330 (19)	0.037 (2)	0.0147 (16)	0.0079 (16)	0.0037 (15)
C5	0.048 (2)	0.034 (2)	0.0346 (19)	0.0204 (17)	0.0097 (17)	0.0113 (15)
C6	0.0357 (19)	0.038 (2)	0.0319 (19)	0.0159 (16)	0.0114 (16)	0.0127 (15)
C7	0.0343 (19)	0.041 (2)	0.043 (2)	0.0122 (16)	0.0077 (16)	0.0143 (17)
C8	0.045 (2)	0.0317 (19)	0.040 (2)	0.0151 (16)	0.0065 (17)	0.0120 (16)
C9	0.035 (2)	0.0284 (18)	0.055 (2)	0.0098 (15)	0.0019 (17)	0.0097 (17)
C10	0.047 (2)	0.0268 (18)	0.0346 (19)	0.0065 (16)	0.0011 (16)	0.0090 (15)
N1	0.0443 (19)	0.0374 (18)	0.0353 (18)	0.0124 (15)	0.0026 (14)	0.0059 (14)
N2	0.052 (2)	0.0418 (19)	0.0361 (18)	0.0139 (16)	0.0038 (15)	0.0122 (15)
N3	0.081 (3)	0.0366 (19)	0.045 (2)	0.0290 (18)	0.0066 (18)	0.0097 (16)
N4	0.0375 (17)	0.0305 (16)	0.0325 (17)	0.0015 (13)	0.0018 (13)	0.0119 (13)
N5	0.0465 (19)	0.0312 (16)	0.0364 (17)	0.0113 (14)	0.0139 (14)	0.0050 (13)
O1	0.0584 (17)	0.0417 (15)	0.0355 (15)	0.0130 (13)	−0.0024 (13)	0.0163 (12)
O2	0.083 (2)	0.0518 (18)	0.0359 (16)	0.0215 (16)	0.0153 (15)	0.0029 (13)
O3	0.082 (2)	0.0404 (17)	0.070 (2)	−0.0071 (16)	0.0138 (18)	0.0096 (16)
O4	0.140 (4)	0.0456 (19)	0.063 (2)	0.017 (2)	−0.035 (2)	0.0223 (17)
O5	0.082 (2)	0.0534 (19)	0.0335 (15)	0.0252 (17)	−0.0068 (15)	−0.0042 (14)
O6	0.150 (4)	0.0452 (19)	0.061 (2)	0.053 (2)	0.010 (2)	0.0046 (16)
O7	0.142 (4)	0.0444 (19)	0.049 (2)	0.030 (2)	0.005 (2)	0.0194 (15)

### Geometric parameters (Å, °)

**Table:**

C1—C2	1.366 (5)	C9—N5	1.465 (5)
C1—C6	1.454 (5)	C9—C10^ii^	1.504 (5)
C1—N1	1.461 (5)	C9—H9A	0.9700
C2—C3	1.385 (5)	C9—H9B	0.9700
C2—H2	0.9300	C10—N5	1.465 (5)
C3—C4	1.380 (5)	C10—C9^ii^	1.504 (5)
C3—N2	1.441 (5)	C10—H10A	0.9700
C4—C5	1.373 (5)	C10—H10B	0.9700
C4—H4	0.9300	N1—O3	1.210 (4)
C5—C6	1.442 (5)	N1—O2	1.224 (4)
C5—N3	1.464 (5)	N2—O4	1.210 (4)
C6—O1	1.243 (4)	N2—O5	1.221 (4)
C7—N4	1.475 (5)	N3—O6	1.215 (4)
C7—C8	1.508 (5)	N3—O7	1.219 (4)
C7—H7A	0.9700	N4—C8^i^	1.484 (5)
C7—H7B	0.9700	N4—H4A	0.86 (4)
C8—N4^i^	1.484 (5)	N4—H4B	0.86 (4)
C8—H8A	0.9700	N5—H5A	0.86 (4)
C8—H8B	0.9700		
			
C2—C1—C6	125.4 (3)	N5—C9—C10^ii^	110.2 (3)
C2—C1—N1	117.1 (3)	N5—C9—H9A	109.6
C6—C1—N1	117.5 (3)	C10^ii^—C9—H9A	109.6
C1—C2—C3	118.3 (3)	N5—C9—H9B	109.6
C1—C2—H2	120.8	C10^ii^—C9—H9B	109.6
C3—C2—H2	120.8	H9A—C9—H9B	108.1
C4—C3—C2	121.2 (3)	N5—C10—C9^ii^	109.1 (3)
C4—C3—N2	118.7 (3)	N5—C10—H10A	109.9
C2—C3—N2	120.0 (3)	C9^ii^—C10—H10A	109.9
C5—C4—C3	119.5 (3)	N5—C10—H10B	109.9
C5—C4—H4	120.2	C9^ii^—C10—H10B	109.9
C3—C4—H4	120.2	H10A—C10—H10B	108.3
C4—C5—C6	124.3 (3)	O3—N1—O2	122.7 (3)
C4—C5—N3	116.0 (3)	O3—N1—C1	118.9 (3)
C6—C5—N3	119.7 (3)	O2—N1—C1	118.3 (3)
O1—C6—C5	126.4 (3)	O4—N2—O5	122.3 (3)
O1—C6—C1	122.4 (3)	O4—N2—C3	118.7 (3)
C5—C6—C1	111.1 (3)	O5—N2—C3	119.0 (3)
N4—C7—C8	110.9 (3)	O6—N3—O7	122.6 (4)
N4—C7—H7A	109.5	O6—N3—C5	117.9 (3)
C8—C7—H7A	109.5	O7—N3—C5	119.5 (3)
N4—C7—H7B	109.5	C7—N4—C8^i^	112.2 (3)
C8—C7—H7B	109.5	C7—N4—H4A	109 (3)
H7A—C7—H7B	108.0	C8^i^—N4—H4A	109 (3)
N4^i^—C8—C7	110.4 (3)	C7—N4—H4B	114 (3)
N4^i^—C8—H8A	109.6	C8^i^—N4—H4B	102 (3)
C7—C8—H8A	109.6	H4A—N4—H4B	110 (4)
N4^i^—C8—H8B	109.6	C10—N5—C9	110.9 (3)
C7—C8—H8B	109.6	C10—N5—H5A	109 (3)
H8A—C8—H8B	108.1	C9—N5—H5A	109 (3)
			
C6—C1—C2—C3	−2.3 (6)	N4—C7—C8—N4^i^	55.1 (4)
N1—C1—C2—C3	177.5 (3)	C2—C1—N1—O3	44.6 (5)
C1—C2—C3—C4	0.2 (6)	C6—C1—N1—O3	−135.6 (4)
C1—C2—C3—N2	−177.1 (3)	C2—C1—N1—O2	−133.7 (4)
C2—C3—C4—C5	0.2 (6)	C6—C1—N1—O2	46.1 (5)
N2—C3—C4—C5	177.5 (3)	C4—C3—N2—O4	−175.0 (4)
C3—C4—C5—C6	1.6 (6)	C2—C3—N2—O4	2.4 (6)
C3—C4—C5—N3	−178.8 (3)	C4—C3—N2—O5	4.3 (5)
C4—C5—C6—O1	173.8 (4)	C2—C3—N2—O5	−178.4 (4)
N3—C5—C6—O1	−5.7 (6)	C4—C5—N3—O6	−20.6 (6)
C4—C5—C6—C1	−3.2 (5)	C6—C5—N3—O6	159.0 (4)
N3—C5—C6—C1	177.2 (3)	C4—C5—N3—O7	157.2 (4)
C2—C1—C6—O1	−173.5 (4)	C6—C5—N3—O7	−23.2 (6)
N1—C1—C6—O1	6.7 (5)	C8—C7—N4—C8^i^	−56.1 (4)
C2—C1—C6—C5	3.7 (5)	C9^ii^—C10—N5—C9	−58.5 (4)
N1—C1—C6—C5	−176.1 (3)	C10^ii^—C9—N5—C10	59.1 (4)

Symmetry codes: (i) −*x*, −*y*+2, −*z*+1; (ii) −*x*+1, −*y*+3, −*z*+1.

### Hydrogen-bond geometry (Å, °)

**Table:**

*D*—H···*A*	*D*—H	H···*A*	*D*···*A*	*D*—H···*A*
N4—H4A···O1	0.86 (4)	1.95 (4)	2.745 (4)	153 (4)
N4—H4A···O7	0.86 (4)	2.31 (4)	2.870 (5)	123 (3)
N4—H4B···N5	0.86 (4)	1.94 (4)	2.799 (4)	176 (4)
N5—H5A···O2^iii^	0.86 (4)	2.41 (4)	3.153 (5)	145 (4)
C2—H2···O6^iv^	0.93	2.47	3.335 (5)	155
C7—H7B···O1^i^	0.97	2.52	3.211 (5)	128
C8—H8B···O1	0.97	2.60	3.267 (5)	127
C8—H8B···O2	0.97	2.52	3.458 (5)	162
C9—H9A···O5^v^	0.97	2.60	3.272 (5)	127
C10—H10A···O4^vi^	0.97	2.52	3.310 (5)	138

Symmetry codes: (iii) *x*, *y*+1, *z*; (iv) *x*, *y*−1, *z*; (i) −*x*, −*y*+2, −*z*+1; (v) −*x*+1, −*y*+3, −*z*+2; (vi) −*x*+1, −*y*+2, −*z*+2.
